# Analysis of the characteristics and differences in exercise participation among seven occupational categories of female workers in China—a cross-sectional survey of 31 provinces across the country

**DOI:** 10.3389/fpubh.2026.1923105

**Published:** 2026-09-09

**Authors:** Xiao-zhe Qin, Min Li, Zhen-feng Liu, Xin-yue Li

**Affiliations:** 1 School of Physical Education, Henan University, Kaifeng, China; 2 Department of Social Sports, Henan Institute of Physical Education, Zhengzhou, China

**Keywords:** female workers, physical exercise motivation, physical participation, socioecological barriers, women’s physical exercise

## Abstract

**Background:**

Understanding Physical exercise behavioral variations across female occupations is crucial for tailoring interventions and promoting occupational health. This study evaluates physical exercise rates, preferences, motivations, and barriers among Chinese working women.

**Methods:**

A cross-sectional survey was conducted with 3,740 female participants across seven occupational categories in 31 Chinese provinces. Physical exercise behavior was assessed using the Physical Exercise Rating Scale; exercise types were classified according to China’s National Fitness Guidelines; exercise motivation was developed based on Self-Determination Theory; and multi-level participation barriers were measured under the socioecological framework for women’s physical activity. Data were analyzed using ANOVA, Chi-square tests, and multiple linear regression.

**Results:**

(1) Exercise intensity per session was generally low, with significant occupational differences in intensity (*F* = 3.818, *p* < 0.01), duration (*F* = 5.222, *p* < 0.001), and weekly frequency (*F* = 8.561, *p* < 0.001). (2) Leisure activities dominated, though preferences varied significantly by occupation (all *p* < 0.01): civil servants and Educators favored team sports and traditional national sports; migrant workers highly preferred dance and gymnastics; and corporate employees led in outdoor sports, whereas farmers led in strength training. (3) Physical fitness was a universal motivator with significant occupational differences (*p* < 0.01), whereas fat reduction, though widely endorsed, did not differ significantly across occupations (*p* > 0.05). Corporate employees showed the weakest motivations for socialization, stress relief, and family time, whereas migrant workers exercised primarily for socialization. (4) Lack of perseverance constrained most women. Migrant workers faced only time limitations (*β* = −0.365, *p* = 0.001), while civil servants uniquely encountered policy barriers (*β* = 0.069, *p* < 0.05). The urban built environment positively promoted physical exercise across all urban occupations.

**Conclusion:**

Occupational resources, labor types, and workplace cultures stratify physical exercise participation among Chinese women. Public-sector employees enjoy institutional resource advantages, while rural and migrant women face structural deficits. We should start from the workplace and promote differentiated and compensatory public sports services based on occupational characteristics.

## 1 Introduction

The workplace is a key social factor determining health inequalities among populations (1). The sedentary lifestyle, high socio-psychological burden, and institutionalized encroachment on working hours inherent in modern occupational environments are becoming risk factors for chronic non-communicable diseases and musculoskeletal disorders among female workers (2). Physical exercise, as a highly cost-effective proactive health intervention mechanism, has irreplaceable public health value in alleviating occupational stress, reducing chronic disease risks, and improving workforce sustainability for women (3, 4). In research practice, physical exercise is neither interchangeable with the broader concept of physical activity nor equivalent to the narrower category of sports. Physical activity functions as an umbrella term covering all energy-consuming bodily movements generated by skeletal muscles, spanning occupational labor, housework, daily commuting and leisure activities. As a deliberate subset of leisure-time physical activity, physical exercise refers to planned, structured and repetitive bodily movement performed specifically to improve or maintain physical fitness and health status, which is the core variable examined in this study (5). By comparison, sports represent a specialized, rule-governed form of exercise with competitive attributes and standardized participation frameworks (6). Physical exercise is not an individual behavior but is structurally constrained by the occupational level and organizational environment (7). Different occupational types imply distinct occupational characteristics, reflected not only in objective working hours and physical exertion but also in organizational health culture and resource accessibility (8). These differentiated occupational characteristics constitute structural barriers for women in different occupations regarding physical exercise thresholds, activity preferences, and behavioral habits (9, 10).

There are occupational differences in physical exercise, which have been confirmed in previous studies. A systematic review of 131 European studies by Gidlow et al. showed that 68% of the studies believed that the socio-economic status brought by a profession was positively correlated with physical exercise, with higher rates of physical exercise among women engaged in professional technical and managerial work than among manual laborers and service workers (11). In China, a nationwide survey of 13,395 female workers found that only 15.6% of the working population regularly engage in physical exercise, and the proportion of women is lower than that of men (12). However, current research has not explored the differences in physical exercise among women of different professions. China’s rapid economic transformation and the rise of modern service industries have fostered a highly diverse and differentiated occupational landscape for women, providing a unique sample for observing differences in health behaviors within occupational environments. Therefore, this study, based on a nationwide cross-sectional survey, aims to systematically understand the objective characteristics of exercise participation rates, intensity, exercise preferences, and barriers among women in different Occupations in China. It aims to provide a scientific basis for public health departments to formulate precise women’s health intervention policies “based on occupational characteristics.”

## 2 Materials and methods

### 2.1 Study design and data source

This study adopted a cross-sectional study design. Fieldwork was conducted from June 2025 to September 2025 across 31 provincial-level administrative regions in mainland China. This research is a sub-study derived from a large nationwide survey on women’s physical exercise. All data were collected through primary investigation for the overarching research project. The analytical sample of the current study was obtained by excluding retirees, students and participants with unidentifiable occupational status from the full survey dataset. This study does not constitute a secondary analysis of previously published or external datasets.

### 2.2 Sampling and participants

Based on the occupational stratification framework proposed in the Research Report on Social Strata in Contemporary China, this study focuses on female populations across seven distinct occupational categories: corporate employees, private business owners, educators, civil servants, farmers, migrant workers, and healthcare workers (13).

#### 2.2.1 Sampling strategy

A mixed stratified sampling approach was adopted, covering both onsite and online survey modes.

Online survey: Convenience sampling was implemented via the Wenjuanxing online platform, to achieve full geographical coverage across 31 provincial-level administrative regions.

Onsite field survey: A two-layer stratified sampling strategy based on geographical division and economic gradient was applied. In the first layer, the whole country was divided into eastern, central and western regions in accordance with the national standard classification. In the second layer, representative cities with high, medium and low development gradients were selected from each region, with consideration of regional economic development level, urbanization rate, population structure and geographical characteristics. Finally, 11 cities were included in the onsite survey: 6 cities in eastern China (Beijing, Shanghai, Guangzhou, Changzhou, Hangzhou, Harbin), 3 cities in central China (Wuhan, Zhengzhou, Taiyuan), and 2 cities in western China (Chengdu, Xi’an). Data from both online and onsite surveys were pooled to form the full survey dataset.

#### 2.2.2 Sample size determination

The sample size was not pre-determined by *a priori* power calculation targeting different occupational groups. The final analytical sample was derived from the cleaned valid questionnaires of the full survey, after excluding participants with unidentifiable occupation, students and retirees. A total of 7,777 questionnaires were distributed in the large-scale survey, yielding 6,918 valid responses (an effective rate of 89%). For the current study, questionnaires from retirees, students, and individuals with unidentifiable occupations were excluded, leaving a final analytical sample of 3,740 participants. For the large nationwide survey, the inclusion criteria were: (1) female sex; (2) aged 14 years or older; (3) residing in mainland China; and (4) provision of informed consent. The exclusion criteria were: (1) incomplete questionnaires with more than 20% missing items; (2) patterned or careless responses (e.g., straight-line responding); and (3) failure to complete the informed consent procedure. For the current sub-study, the inclusion criteria were further restricted to female respondents with clear occupational information and stable paid jobs, and the exclusion criteria additionally comprised full-time students, retirees, those without fixed occupations, and samples with missing occupational information.

#### 2.2.3 Ethical considerations

This study was granted an exemption from ethical review by the Institutional Review Board of Henan University, as it only involved anonymous questionnaire surveys without invasive physiological sampling or collection of sensitive private information. All procedures were performed in accordance with the Declaration of Helsinki. An informed consent statement was presented on the first page of the questionnaire, which fully explained the research purpose and rules of anonymity and confidentiality to participants. All participants joined the study voluntarily after being fully informed, and could terminate questionnaire completion at any time. In China, the legal age of majority is 18 years. Given that 51 participants (1.40%) were aged 14–17 years, informed consent for these minor participants was obtained from their parents or legal guardians, alongside the participants’ own assent, in accordance with Chinese research ethics regulations governing research involving minors.

### 2.3 Measurements

The questionnaire consisted of five sections: basic demographic information, physical exercise behavior, types of physical exercise, physical exercise motivation, and barriers to physical exercise participation. Content validity was evaluated by a panel of experts in sports science, exercise psychology, and public health, who reviewed the relevance, clarity, and comprehensiveness of items across all sections; minor revisions to item wording were made based on expert feedback. Before formal large-scale distribution, a pilot study was conducted among 30 female workers to test item readability, logical flow, and completion time. Based on pilot feedback, wording of several items was further revised for clarity. After formal data collection, a random subsample of 486 valid responses was drawn from the full survey dataset to evaluate the psychometric properties of the barrier measurement scale. Exploratory factor analysis and confirmatory factor analysis were conducted on this subsample.

Physical exercise behavior and overall exercise volume were measured using Liang Deqing’s Physical Activity Rating Scale (PARS-3), which consists of three items assessing exercise intensity, single-session duration, and weekly exercise frequency. Participants were instructed to recall their physical exercise performance over the past week. Each item was scored on a 5-point scale. For exercise intensity, scores from 1 to 5 corresponded to mild exercise, low-intensity non-strenuous exercise, moderate-intensity relatively strenuous exercise, high-intensity exercise with rapid breathing and heavy sweating, and maximum-endurance exhaustive exercise, respectively. For single-session duration, the five levels represented <10 min, 10–20 min, 20–30 min, 30–60 min, and >60 min. For weekly frequency, the categories were 0 times per week, 1–2 times per week, 3 times per week, 4–5 times per week, and 6–7 times per week. Total exercise volume was calculated as the product of intensity, duration, and frequency scores, yielding a total score ranging from 1 to 125. Higher total scores indicated greater overall physical exercise volume among female participants. The scale has a test–retest reliability coefficient of 0.82, indicating satisfactory temporal stability (14).

Classification of physical exercise types was formulated based on the framework of China’s first National Fitness Guidelines, which categorizes physical activities into aerobic exercise, strength training, ball games, and traditional ethnic sport (15). Building on this framework, the present study further refined the classification by designing questionnaire items based on specific exercise program. Seven categories were included: racquet sports, team sports, leisure and recreational activities,outdoor sports, strength training, dance and gymnastics, traditional national sports. Each exercise type was coded as a binary variable, with 0 indicating no participation and 1 indicating participation in that exercise type. The proportion of participants engaging in each type was presented as a percentage.

The Physical exercise motivation section was developed based on Self-Determination Theory and contained nine items covering intrinsic and extrinsic motivations. Self-Determination Theory (SDT), proposed by Deci, is a motivational framework that distinguishes between intrinsic motivation (engaging in an activity for its inherent satisfaction and enjoyment) and extrinsic motivation (engaging in an activity to achieve separable outcomes), and posits that autonomous and self-regulated forms of motivation are associated with more sustained behavior adherence and better psychological wellbeing (16). The motivational dimensions included intrinsic motivation factors such as personal interest and habits, athletic skill development, and pastime, and extrinsic motivation factors such as physical fitness improvement, weight loss and lipid reduction, social interaction, stress relief, disease prevention, and family companionship. Both the Physical exercise type and motivation sections adopted binary coding: 0 indicated no participation or absence of a given motivation, while 1 indicated participation or presence of the corresponding motivation. The proportion of participants engaging in each type was presented as a percentage.

The barrier measurement section drew on the socioecological model of Physical exercise for women and girls released by the Canadian Association for the Advancement of Women and Girls in Sport and Physical Activity (17). Survey items were constructed across four layers of the socioecological framework: individual, interpersonal, societal, and policy-level factors, with a total of 50 items. All barrier items were scored on a 5-point Likert scale, where 1 = no impact at all, 5 = extreme impact, and intermediate values represented varying degrees of perceived influence between the two endpoints.

Internal consistency was tested using Cronbach’s alpha coefficients to ensure reliability. The Cronbach’s alpha for the overall barrier scale was 0.899, with specific dimensions showing high reliability: personal factors (0.855), interpersonal factors (0.854), social factors (0.811), and policy factors (0.885). Structural validity was evaluated through Exploratory Factor Analysis (EFA) and Confirmatory Factor Analysis (CFA). EFA using principal component analysis with varimax rotation extracted 14 principal components, explaining 75.66% of the total variance (KMO = 0.940, *p* = 0.001), indicating excellent structural validity. CFA was subsequently performed using Amos 24.0. The 14 primary factors extracted from EFA were defined as first-order factors, while ‘personal,’ ‘interpersonal,’ ‘social,’ and ‘policy’ factors served as second-order factors. Specifically, ‘personal objective,’ ‘personal time,’ ‘personal image,’ ‘personal physiological,’ ‘personal perseverance,’ and ‘personal psychological’ factors were first-order factors for ‘personal factors.’ ‘Interpersonal participation,’ ‘exercise-related problems,’ and ‘interpersonal support’ were first-order factors for ‘interpersonal factors.’ ‘Social sports atmosphere,’ ‘built environment,’ ‘natural environment,’ and ‘exercise climate’ were first-order factors for ‘social factors,’ while ‘policy factors’ directly served as a second-order factor. The final model fit indices for the second-order model demonstrated good structural validity: *χ*^2^/df = 2.930, RMSEA = 0.051, CFI = 0.906, IFI = 0.907, and TLI = 0.900.

### 2.4 Statistical analysis

Normality was assessed by the Shapiro–Wilk test and homogeneity of variances by Levene’s test prior to parametric analyses. One-way Analysis of Variance (ANOVA) and Chi-square (*χ*^2^) tests were employed to examine the differences in physical exercise duration, intensity, frequency, activity types, and motivations across female workers of different occupations. Multiple linear regression models were used to examine the associations between exercise volume and multi-level exercise barriers, with total physical exercise volume as the dependent variable and barrier factor scores as the independent variables. Pearson product–moment correlation analysis was performed to examine the bivariate associations between the four barrier dimensions (personal, interpersonal, social, and policy factors) and total physical exercise volume. All statistical analyses were performed using SPSS 26.0. The significance level was set at *p* < 0.05 (two-tailed).

## 3 Results

### 3.1 Demographic characteristics

Table 1 presents the demographic characteristics of the 3,740 participants. The majority were aged 26–35 years (38.50%), followed by 36–45 years (26.90%). Most participants had a junior college or bachelor’s degree (63.30%), and 22.00% held a postgraduate degree or above. Corporate employees accounted for the largest proportion (41.80%), followed by civil servants (26.50%). Over half were married with children (55.90%), and 33.70% were unmarried. The annual individual income was mainly between 50,000 and 99,999 RMB (34.80%), with 22.00% reporting an income of 100,000 RMB or above.

**Table 1:** Demographic information of the participants.

Demographic variable	Category	Frequency (*n*)	Percentage (%)
Age	14–17	51	1.40
	18–25	670	17.90
	26–35	1,439	38.50
	36–45	1,006	26.90
	46–55	459	12.30
	56 and above	115	3.10
Education level	Lower secondary education or below	131	3.50
	Upper secondary/vocational education	417	11.10
	Junior college or bachelor’s degree	2,369	63.30
	Postgraduate degree or above	822	22.00
Occupation	Corporate employees	1,562	41.80
	Private business owners	300	8.00
	Educators	318	8.50
	Civil servants	990	26.50
	Farmers	175	4.70
	Migrant workers	163	4.40
	Healthcare workers	232	6.20
Marital and family status	Unmarried	1,262	33.70
	Married, without children	302	8.10
	Married, with children	2,091	55.90
	Divorced, without children	68	1.80
	Divorced, with children	17	0.50
Annual individual income	Below 20,000 RMB	366	9.80
	20,000–34,999 RMB	529	14.10
	35,000–49,999 RMB	721	19.30
	50,000–99,999 RMB	1,303	34.80
	100,000 RMB and above	821	22.00

### 3.2 Descriptive statistics of main variables

Table 2 presents the descriptive statistics for the main study variables. The mean scores for exercise intensity, duration, and frequency were 2.01 ± 0.96, 3.47 ± 1.57, and 3.20 ± 1.78, respectively. The most frequently reported exercise type was leisure and recreational activities (60.5%), followed by racquet sports (29.4%) and strength training (19.2%), while traditional national sports had the lowest participation rate (3.2%). The leading motivation for exercise was physical fitness (77.6%), followed by fat reduction (57.4%) and stress regulation (42.9%). Among the 14 barrier items, built environment (3.85 ± 0.99) and natural environment (3.73 ± 1.02) scored highest, whereas personal image (2.28 ± 1.07) and social sports atmosphere (2.32 ± 0.92) scored lowest.

**Table 2:** Descriptive statistics of main study variables.

Variable	Item	Mean ± SD	No	Yes
Physical exercise behavior	Exercise intensity per session	2.01 ± 0.96	–	–
	Exercise duration per session	3.47 ± 1.57	–	–
	Exercise frequency per week	3.20 ± 1.78	–	–
Physical exercise types	Racquet sports	–	70.6	29.4
	Team sports	–	92.5	7.5
	Leisure and recreational activities	–	39.5	60.5
	Outdoor sports	–	85.9	14.1
	Strength training	–	80.8	19.2
	Dance and gymnastics	–	84.2	15.8
	Traditional national sports	–	96.8	3.2
Physical exercise motivations	Physical fitness	–	22.4	77.6
	fat reduction	–	42.6	57.4
	Socialization	–	78.3	21.7
	Interest and habit	–	71.2	28.8
	Stress regulation	–	57.1	42.9
	Disease prevention	–	73.3	26.7
	Sport skill improvement	–	86.6	13.4
	Family companionship	–	81.9	18.1
	Leisure and recreation	–	73.0	27.0
Physical exercise barriers	Personal objective	2.71 ± 0.91	–	–
	Personal time	3.34 ± 1.10	–	–
	Personal image	2.28 ± 1.07	–	–
	Personal physiological	3.58 ± 1.08	–	–
	Personal perseverance	3.05 ± 1.03	–	–
	Personal psychological	2.96 ± 1.08	–	–
	Interpersonal participation	2.68 ± 1.12	–	–
	Exercise-related problems	2.46 ± 0.97	–	–
	Interpersonal support	2.59 ± 1.00	–	–
	Social sports atmosphere	2.32 ± 0.92	–	–
	Built environment	3.85 ± 0.99	–	–
	Natural environment	3.73 ± 1.02	–	–
	Exercise climate	2.98 ± 1.11	–	–
	Policy factors	2.75 ± 1.06	–	–

### 3.3 Characteristics of physical exercise behavior among Chinese women across different occupations

The Analysis of Variance (ANOVA) revealed that the between-group effects for all three exercise metrics reached highly significant levels: intensity per session (*F* = 3.818, *p* < 0.01), duration per session (*F* = 5.222, *p* < 0.001), and weekly frequency (*F* = 8.561, *p* < 0.001). These findings confirm robust and significant group-level differences in exercise intensity, duration per session, and weekly frequency among women of different occupations. This demonstrates that occupational identity serves as a crucial social stratification variable explaining the differentiation in female physical exercise behavior (Table 3).

**Table 3:** One-way ANOVA of physical exercise behaviors among female workers across different occupations.

Exercise behavior	Corporate employees	Private business owners	Educators	Civil servants	Farmers	Migrant workers	Healthcare workers	*F*	*p*	df₁	df₂	Partial *η*^2^
Exercise intensity per session	2.01 ± 0.95ab	2.04 ± 0.97ab	2.16 ± 1.00b	2.03 ± 0.99ab	2.06 ± 0.89ab	1.83 ± 0.83a	1.83 ± 0.93a	3.818	0.001	6	3,733	**0.006**
Exercise duration per session	3.36 ± 1.52a	3.42 ± 1.60a	3.36 ± 1.46a	3.56 ± 1.61a	3.63 ± 1.56b	3.95 ± 1.74b	3.62 ± 1.67b	5.222	<0.001	6	3,733	**0.008**
Exercise frequency per week	3.06 ± 1.69a	3.07 ± 1.84a	2.98 ± 1.69a	3.38 ± 1.74bc	3.51 ± 2.02bc	3.82 ± 2.08c	3.17 ± 1.98b	8.561	<0.001	6	3,733	**0.014**

Data are presented as mean ± standard deviation. Different lowercase superscript letters (a, b, c) within the same row indicate statistically significant differences between occupational groups based on post-hoc pairwise comparisons (*p* < 0.05); groups sharing the same letter do not differ significantly from each other.

Further post-hoc tests indicated that regarding exercise intensity, educators (2.16 ± 1.00) scored significantly higher than migrant workers (1.83 ± 0.83) and healthcare workers (1.83 ± 0.93), with the latter two groups exhibiting the lowest exercise workload per session across all occupations. In terms of duration per session, migrant workers (3.95 ± 1.74) reported significantly longer sessions than corporate employees, private business owners, educators, and civil servants, while farmers (3.63 ± 1.56), and healthcare workers (3.62 ± 1.67) showed relatively longer but not statistically different durations.

Regarding weekly frequency, migrant workers (3.82 ± 2.08) and farmers (3.51 ± 2.02) exercised most frequently, whereas the weekly exercise frequencies of educators (2.98 ± 1.69), corporate employees (3.06 ± 1.69), and private business owners (3.07 ± 1.84) were significantly lower. Full post-hoc pairwise comparisons are presented in Supplementary Table S1.

### 3.4 Distribution and characteristics of physical exercise types among female workers across different occupations

A Pearson Chi-square test of independence was conducted using the seven occupational categories as the grouping variable and the seven physical exercise types as the observed indicators (Table 4). Regarding the overall proportions, leisure and recreational activities—primarily walking and brisk walking—constituted the most common exercise types among all women. Furthermore, the independence test revealed highly significant occupational disparities across all exercise types (*p* < 0.01), indicating that occupational identity exerts a significant influence on women’s choice of sports.

**Table 4:** Distribution and chi-square test of physical exercise types among female workers across different occupations.

Exercise type	Corporate employees	Private business owners	Educators	Civil servants	Farmers	Migrant workers	Healthcare workers	*χ* ^2^	*p*	Cramer’s *V*
Racquet sports	28.55%	37.00%	37.74%	29.19%	25.14%	18.40%	26.29%	31.628	<0.001	0.092
Team sports	4.93%	7.00%	13.52%	11.01%	6.29%	4.29%	5.60%	53.09	<0.001	0.119
Leisure and recreational activities	66.01%	58.67%	71.07%	55.35%	52.57%	41.10%	52.59%	82.397	<0.001	0.148
Outdoor sports	17.61%	14.00%	13.84%	10.51%	9.71%	9.20%	13.36%	32.51	<0.001	0.093
Strength training	21.06%	21.00%	18.24%	17.07%	28.57%	6.75%	16.38%	34.592	<0.001	0.096
Dance and gymnastics	12.55%	16.33%	14.78%	15.76%	17.14%	47.24%	15.95%	133.876	<0.001	0.189
Traditional national sports	1.92%	3.67%	5.35%	4.44%	2.86%	3.68%	3.02%	18.307	0.006	0.070

To further pinpoint the distinct characteristics of exercise preferences across specific occupational groups, post-hoc comparisons were performed using the adjusted standardized residuals matrix (Table 5), with an absolute value greater than 2 (|*R*| > 2) serving as the criterion for a significant deviation from the mean. The results are as follows:

- Racquet sports (Small ball games): Educators (*R* = 3.4) and private business owners (*R* = 3.0) exhibited significantly higher participation rates than the average, whereas Civil servants (*R* = −2.0) and migrant workers (*R* = −3.1) showed significantly lower rates.
- Team sports (Large ball games): Civil servants (*R* = 4.9) and educators (*R* = 4.2) presented prominent positive residuals, indicating a stronger preference for competitive team sports among women in the public sector; conversely, corporate employees reported the lowest propensity to participate, significantly below the mean (*R* = −5.1).
- Leisure and recreational activities: Corporate employees (*R* = 5.9) and educators (*R* = 4.0) selected these activities at a significantly higher rate, while migrant workers demonstrated the greatest negative deviation (*R* = −5.2).
- Outdoor sports: Only corporate employees displayed a significantly positive residual (*R* = 5.2), highlighting a pronounced demand for autonomous outdoor activities among this white-collar group; in contrast, Civil servants showed a significantly lower participation rate (*R* = −3.8).
- Strength training: Farmers (*R* = 3.2) and corporate employees (*R* = 2.5) had significantly higher participation, while migrant workers (*R* = −4.1) and Civil servants (*R* = −2.0) were significantly below the average.
- Dance and gymnastics: Migrant workers exhibited an exceptionally strong positive deviation, with a residual as high as 11.2, identifying dance and gymnastics as the core distinguishing exercise type for this group; corporate employees rarely participated in these group-based movements (*R* = −4.7).
- Traditional national sports: Civil servants (*R* = 2.6) and educators (*R* = 2.3) showed significantly positive residuals, suggesting that public-sector women are more receptive to traditional Chinese wellness exercises (e.g., Tai Chi, Baduanjin); meanwhile, corporate employees participated at a rate significantly below the average (*R* = −3.8).

**Table 5:** Adjusted standardized residuals matrix of exercise types among female workers across different occupations.

Exercise type	Corporate employees	Private business owners	Educators	Civil servants	Farmers	Migrant workers	Healthcare workers
Racquet sports	−1.0	3.0*	3.4*	−2*	−1.3	−3.1*	−1.1
Team sports	−5.1*	−0.4	4.2*	4.9*	−0.6	−1.6	−1.1
Leisure and recreational activities	5.9*	−0.7	4*	−3.8*	−2.2*	−5.2*	−2.5*
Outdoor sports	5.2*	−0.1	−0.2	−3.8*	−1.7	−1.8	−0.3
Strength training	2.5*	0.8	−0.5	−2*	3.2*	−4.1*	−1.1
Dance and gymnastics	−4.7*	0.2	−0.5	−0.1	0.5	11.2*	0.1
Traditional national sports	−3.8*	0.5	2.3*	2.6*	−0.3	0.3	−0.2

* indicates adjusted standardized residuals with absolute values ≥ 2, representing a significant deviation from the expected frequency at *p* < 0.05.

### 3.5 Physical exercise motivations among female workers across different occupations

Using occupational categories as the grouping variable, a Pearson Chi-square test of independence was performed across the nine physical exercise motivations among female workers (Table 6). The overall analysis revealed that three motivations—fat reduction, disease prevention, and sport skill improvement—did not reach statistical significance across groups (*p*>0.05). Conversely, the remaining six motivations—physical fitness, social interaction, interest and habit, stress regulation, family companionship, and leisure and recreation—exhibited significant occupational stratification (*p* < 0.05), demonstrating a pronounced differentiation in social, emotional release, family-oriented, and leisure-based exercise pursuits among women in different lines of work.

**Table 6:** Distribution and chi-square test of physical exercise motivations among female workers across different occupations.

Motivation	Corporate employees	Private business owners	Educators	Civil servants	Farmers	Migrant workers	Healthcare workers	*χ* ^2^	*p*	Cramer’s V
Physical fitness	76.76%	76.00%	72.33%	81.41%	80.00%	80.98%	72.41%	19.701	0.003	0.073
Fat reduction	58.07%	56.67%	60.06%	57.07%	58.86%	49.08%	56.90%	6.106	0.411	0.040
Socialization	16.20%	26.33%	22.01%	25.35%	24.57%	33.74%	26.29%	57.044	<0.001	0.123
Interest and habit	24.46%	29.33%	30.82%	32.42%	33.71%	33.74%	32.33%	26.787	<0.001	0.085
Stress regulation	45.77%	37.33%	47.80%	43.74%	40.57%	18.40%	40.09%	53.511	<0.001	0.120
Disease prevention	25.35%	24.33%	25.16%	29.49%	26.86%	29.45%	27.16%	7.3	0.294	0.044
Sport skill improvement	12.10%	16.00%	14.47%	14.85%	12.57%	12.27%	13.36%	6.379	0.382	0.041
Family companionship	14.53%	23.67%	19.81%	20.40%	22.29%	14.11%	22.41%	30.583	<0.001	0.090
Leisure and recreation	25.35%	24.67%	23.58%	30.10%	24.57%	27.61%	33.19%	14.768	0.022	0.063

Regarding the motivational distribution percentages, physical fitness emerged as the primary exercise motivator for all occupational groups, followed by weight/fat reduction as the secondary motivator. To further pinpoint the localized variations within the significantly different motivations, post-hoc comparisons were conducted using the adjusted standardized residuals matrix (Table 7), with|*R*|>2 serving as the threshold for a significant deviation from the mean. The results indicated that:

- Corporate employees presented significantly negative residuals for social interaction (*R* = −6.9), interest and habit (*R* = −5.0), and family companionship (*R* = −4.8), highlighting that the social and family-bonding attributes of exercise are weakest among corporate women.
- Civil servants displayed significantly positive residuals for physical fitness (*R* = 3.3), social interaction (*R* = 3.2), interest and habit (*R* = 2.9), and family companionship (*R* = 2.2), showing that public-sector women concurrently possess health, social, and familial exercise demands.
- Migrant workers exhibited a significantly positive residual for social interaction (*R* = 3.8), but a pronounced negative deviation for stress regulation (*R* = −6.9), reflecting that mobile migrant women exercise primarily for socialization while exhibiting low demand for emotional relief.
- Healthcare workers showed a significantly positive residual for leisure and recreation (*R* = 2.2), highlighting a prominent leisure-oriented pattern in their physical activities.
- Educators demonstrated a significantly negative residual for physical fitness (*R* = −2.4), indicating that compared to other occupations, they are less likely to exercise solely for the purpose of physical conditioning.

**Table 7:** Adjusted standardized residuals matrix of exercise motivations among female workers across different occupations.

Motivation	Corporate employees	Private business owners	Educators	Civil servants	Farmers	Migrant workers	Healthcare workers
Physical fitness	−1.1	−0.7	−2.4*	3.3*	0.8	1.1	−2*
fat reduction	0.7	−0.3	1	−0.3	0.4	−2.2*	−0.2
Socialization	−6.9*	2*	0.1	3.2*	0.9	3.8*	1.7
Interest and habit	−5*	0.2	0.8	2.9*	1.5	1.4	1.2
Stress regulation	3*	−2*	1.8	0.6	−0.6	−6.9*	−0.9
Disease prevention	−1.6	−1	−0.7	2.3*	0	0.8	0.2
Sport skill improvement	−2*	1.4	0.6	1.5	−0.3	−0.5	0
Family companionship	−4.8*	2.6*	0.8	2.2*	1.5	−1.4	1.8
Leisure and recreation	−1.9	−0.9	−1.4	2.6*	−0.7	0.2	2.2*

* indicates adjusted standardized residuals with absolute values ≥ 2, representing a significant deviation from the expected frequency at *p* < 0.05.

### 3.6 Multi-level barriers to physical exercise participation among female workers across different occupations

Correlation analysis (Table 8) indicated that personal factors (*r* = −0.315, *p* < 0.01), interpersonal factors (*r* = −0.209, *p* < 0.01), social factors (*r* = −0.161, *p* < 0.01), and policy factors (*r* = −0.123, *p* < 0.01) were all significantly and negatively correlated with women’s total physical exercise volume. This preliminarily confirms the explanatory power of the socio-ecological model in addressing the barriers to physical exercise participation among Chinese female workers.

**Table 8:** Correlation analysis between physical exercise barriers and physical exercise volume among female workers.

Variable	Exercise volume	personal factors	interpersonal factors	social factors	policy factors
Exercise volume	1				
Personal factors	−0.315**	1			
Interpersonal factors	−0.209**	0.614**	1		
Social factors	−0.161**	0.587**	0.680**	1	
Policy factors	−0.123**	0.432**	0.544**	0.629**	1

** represents *p* < 0.01.

To further precisely identify the core negative constraining mechanisms of different occupational environments on physical exercise volume, stepwise multiple linear regression models were constructed by occupation, using the 14 first-order barrier factors as independent variables and physical exercise volume as the dependent variable (Table 9). All regression models were statistically significant (*p* < 0.05), and multicollinearity diagnostics were well within the safe threshold (VIF < 10). The data revealed that except for migrant workers, who were exclusively affected by the single rigid constraint of ‘personal time’ (*β* = −0.365, *t* = −4.967, *p* = 0.001), ‘personal perseverance’ emerged as the core predictor across all other occupations, indicating that individual volition is the primary factor influencing Chinese women’s physical exercise. Additionally, ‘built environment’ was another pervasive factor, positively predicting the physical exercise of corporate employees, educators, civil servants, and healthcare workers. Beyond these shared characteristics, the influencing factors varied across occupations. Corporate employees and civil servants encountered the highest number of obstacles, spanning personal, interpersonal, and social dimensions; notably, civil servants were the unique group significantly affected by ‘policy barriers’ (*β* = 0.069). Conversely, farmers and migrant workers faced the fewest constraints, which were limited strictly to the personal dimension. Finally, the core barriers for healthcare workers and educators resided at the personal and environmental levels, whereas the impediments for corporate employees were concentrated at the personal and interpersonal levels.

**Table 9:** Multiple linear regression models of barriers to physical exercise among female workers across different occupations.

Occupation	Variable	Unstandardized coefficients		*β*	*t*	*p*	95% CI	VIF	*R* ^2^	Adjusted *R*^2^	*F*
		*B*	SE								
Corporate employees	(Constant)	43.462	2.149		20.226	<0.001	[39.247, 47.677]		0.120	0.117	35.34
	Personal perseverance	−1.318	0.213	−0.198	−6.194	<0.001	[−1.736, −0.900]	1.81			
	Personal objective	−0.348	0.162	−0.072	−2.151	0.032	[−0.666, −0.030]	1.98			
	Personal time	−0.680	0.232	−0.085	−2.930	0.003	[−1.135, −0.225]	1.49			
	Interpersonal participation	−0.486	0.206	−0.064	−2.355	0.019	[−0.890, −0.082]	1.31			
	Built environment	0.486	0.109	0.130	4.466	<0.001	[0.272, 0.700]	1.50			
	Natural environment	−0.799	0.183	−0.132	−4.380	<0.001	[−1.158, −0.440]	1.60			
Private business owners	(Constant)	38.587	3.393		11.373	<0.001	[31.910, 45.264]		0.090	0.084	14.69
	Personal perseverance	−1.637	0.364	−0.267	−4.494	<0.001	[−2.353, −0.921]	1.15			
	Exercise-related problems	−0.585	0.254	−0.137	−2.307	0.022	[−1.085, −0.085]	1.15			
Educators	(Constant)	30.332	4.932		6.15	<0.001	[20.628, 40.036]		0.160	0.149	14.90
	Personal perseverance	−1.846	0.412	−0.277	−4.485	<0.001	[−2.657, −1.035]	1.42			
	Natural environment	−1.237	0.355	−0.222	−3.484	0.001	[−1.935, −0.539]	1.51			
	Built environment	0.601	0.237	0.159	2.540	0.012	[0.135, 1.067]	1.46			
	Personal physiological	1.082	0.457	0.144	2.370	0.018	[0.183, 1.981]	1.38			
Civil servants	(Constant)	43.616	2.758		15.812	<0.001	[38.204, 49.028]		0.170	0.164	28.73
	Personal perseverance	−2.439	0.291	−0.333	−8.386	<0.001	[−3.010, −1.868]	1.87			
	Interpersonal support	−1.199	0.243	−0.180	−4.932	<0.001	[−1.676, −0.722]	1.58			
	Built environment	0.640	0.149	0.167	4.289	<0.001	[0.348, 0.932]	1.79			
	Personal time	−0.943	0.316	−0.106	−2.986	0.003	[−1.563, −0.323]	1.49			
	Personal physiological	0.958	0.326	0.102	2.942	0.003	[0.318, 1.598]	1.42			
	Natural environment	−0.531	0.249	−0.085	−2.137	0.033	[−1.020, −0.042]	1.87			
	Policy factors	0.177	0.090	0.069	1.967	0.049	[0.000, 0.354]	1.46			
Farmers	(Constant)	44.752	4.419		10.128	<0.001	[36.030, 53.474]		0.146	0.141	29.56
	Personal perseverance	−2.532	0.466	−0.382	−5.437	<0.001	[−3.452, −1.612]	1.00			
Migrant workers	(Constant)	38.971	3.637		10.716	<0.001	[31.789, 46.153]		0.133	0.127	24.67
	Personal time	−2.960	0.596	−0.365	−4.967	<0.001	[−4.137, −1.783]	1.00			
Healthcare workers	(Constant)	43.013	5.252		8.190	<0.001	[32.664, 53.362]		0.150	0.135	10.01
	Personal perseverance	−1.895	0.477	−0.283	−3.976	<0.001	[−2.835, −0.955]	1.35			
	Personal time	−1.720	0.639	−0.193	−2.690	0.008	[−2.979, −0.461]	1.37			
	Built environment	0.640	0.247	0.176	2.594	0.010	[0.153, 1.127]	1.23			
	Sports atmosphere	−1.221	0.583	−0.142	−2.095	0.037	[−2.370, −0.072]	1.23			

## 4 Discussion

Based on a large cross-sectional sample covering 31 provinces in China, this study analyses the group stratification characteristics of seven occupational categories of female workers regarding their physical exercise behaviors, sport preferences, intrinsic motivations, and multi-level barriers. The findings indicate that the physical exercise characteristics of Chinese female workers exhibit both universal commonalities and distinct differences.

Integrating the data from all seven occupational categories reveals a stable and uniform pattern of physical participation across groups. In terms of physical exercise behavior, the exercise intensity per session for all female workers was generally low, with light-intensity activities predominating across all occupational groups. This reflects a pronounced deficiency in the awareness of the health benefits associated with different exercise intensities among Chinese female workers (18). Regarding exercise preferences, leisure activities such as walking and brisk walking emerged as the mainstream exercise modality for all women, highlighting a prominent homogeneity in exercise selection and a lack of diverse participation. However, this result also confirms that public exercise service initiatives, such as the construction of fitness trails and urban exercise parks in China, have achieved tangible success (19). In terms of motivations, physical fitness and fat reduction constituted the two core drivers, concurrently reflecting contemporary female workers’ dual pursuit of physical health maintenance and body aesthetics (20). The regression results for barriers show that a lack of personal perseverance is the primary constraint inhibiting long-term adherence across all groups. This characteristic indicates that physical exercise has not yet been integrated into women’s daily routines; rather, it remains a conscious health behavior maintained solely through self-discipline, failing to form a spontaneous, normalized lifestyle. Overall, a gap still exists between the general physical exercise participation behaviors of current Chinese women and those in Western developed nations (21, 22), which this study empirically validates through the lens of specific occupational contexts.

Regarding differences, the physical exercise characteristics of women in various occupations reflect not only the impact of job attributes on individuals but also the cultural temperaments and social stratification features generated by different Occupations. Civil servants and educators demonstrated significantly higher participation in team sports compared to other occupational groups. This structural disparity profoundly reveals the shaping role of ‘organizational and institutional support’ on individual exercise behavior within specific Chinese occupational fields (23). In Chinese community sports, team sports have long faced a weak grassroots foundation and a scarcity of public venue resources (24). For female groups in particular, the practical hurdles of ‘difficulty in team formation and lack of space’ are more severe than for men, often leading to a lack of female participation in major team sports (25). However, this study highlights the high participation of these two groups, which can be attributed to government agencies and educational institutions functioning as typical ‘danwei’ (work unit) platforms within the institutional system, effectively leveraging resource compensation mechanisms. Educators have access to well-established sports infrastructures at their workplaces (e.g., school gymnasiums), which significantly lowers the physical barrier to participation (26). Civil servants rely on normalized employee tournaments and organizational networks within the institutional system, thereby gaining high-frequency, structured opportunities for social exercise (27). Consequently, the intersection of workplace spatial convenience and organizational mobilization constitutes a unique ‘institutional dividend’ for team sports participation among these two categories of female workers. With sufficient sports resources, the exercise motivations and barriers of women become increasingly diverse. For instance, civil servants exhibited a contradictory phenomenon of both high motivation and high barriers, which this study interprets as a developmental stage wherein individuals, having met their basic needs, pursue high-quality exercise experiences based on unique personal demands.

Farmers and migrant workers represent occupational groups with weaker access to physical participation resources, facing structural barriers to their physical exercise involvement. Previous studies have shown that rural sports development in China lags far behind urban areas, with most farmers lacking sport awareness, behaviors, and necessary conditions (28). This study presents a contrasting result: farmers’ exercise duration per session was significantly higher than that of other groups, and their frequency of participation in strength training was also higher. Combined with field survey experiences, this study supports previous conclusions while offering a contextualized understanding of the current survey results. Interacting with farmers during the fieldwork revealed that they indeed lacked structured exercise habits, foundational sport knowledge, and infrastructure. However, they possessed a clear health consciousness, perceiving daily agricultural labor as a form of bodily exercise that serves the purpose of health promotion through physical activity. This explains the discrepancy with previous literature and reflects a shift in farmers’ awareness regarding movement-driven health promotion. However, agricultural labor is essentially occupational physical activity (OPA) rather than leisure-time exercise (LTPA) (29). The PARS-3 relies on self-report, and farmers may have subsumed prolonged farming tasks under “exercise,” inflating their duration and strength-type scores. This resonates with the “physical activity paradox”: high OPA, unlike LTPA, does not yield equivalent cardiovascular benefits and may even be detrimental due to insufficient recovery and repetitive loading (30). Thus, farmers’ seemingly higher exercise scores likely reflect a measurement artifact rather than genuinely adequate structured exercise, underscoring the need for objective measures to disentangle OPA from LTPA in rural populations.

Prior research suggests that urban integration challenges serve as an external cause for the insufficient physical exercise resources among migrant workers (31). The results of this study further corroborate and deepen this perspective. Migrant workers’ motivation for ‘social interaction’ was significantly higher than that of other groups, indicating an intrinsic psychological demand for social integration (32). Their participation in dance and gymnastics (typically referring to square dancing) was substantially higher than other groups, illustrating their extremely low occupancy of urban public sports facilities. Square dancing requires minimal venue space, needing only a small clearing and a speaker to be conducted anytime and anywhere, making it a highly popular exercise among contemporary women; however, a lack of management has generated numerous social governance issues (33). Additionally, occupational characteristics are vital factors influencing the physical exercise participation of farmers and migrant workers (34). The barriers for these two groups exist strictly at the personal level, indicating that populations engaged in manual labor experience higher occupational energy expenditure and longer working hours, which subsequently lower their willingness to participate and constrain their exercise initiatives.

While educators, corporate employees, and healthcare workers do not display obvious advantages or disadvantages in resource ownership, their physical exercise behaviors similarly reflect distinct occupational hallmarks. Educators’ exercise intensity per session was significantly higher than that of other groups, indicating that within China’s high-pressure educational evaluation system, high-intensity exercise serves as a vital outlet for teachers to vent occupational stress and adjust their personal states (35). Corporate employees exhibited a clear clustering effect in exercise types, with participation in leisure, outdoor, and strength sports significantly higher than in other categories, while their involvement in remaining sport types was significantly below the average. Outdoor sports are currently being vigorously developed by Chinese industrial policies, while strength training primarily occurs in muscle-sculpting gym settings, indicating that corporate employees represent a trendier segment in their choice of exercise modalities (36). Healthcare workers exhibited significantly longer exercise durations per session but significantly lower motivations for weight/fat reduction. This suggests that given their professional knowledge background, they focus more on achieving health promotion goals through appropriate ‘exercise dosages.’

These findings are broadly consistent with previous research. Large-scale Chinese surveys have shown that physical exercise accounts for only a small fraction of total physical activity among Chinese adults, with farmers and manual workers reporting the lowest leisure-time participation despite high occupational physical demands (37). The predominance of low-intensity leisure activities and the primacy of physical fitness and health motivations across occupational groups echo prior studies of Chinese working populations (38). The universal salience of personal perseverance as a barrier, and the disproportionate time constraints faced by manual and care workers, likewise align with existing evidence on women’s exercise barriers (32). However, the present study extends this literature by systematically comparing seven occupational categories within a single analytical framework and by identifying occupation-specific barrier profiles through stratified regression, revealing, for instance, the paradoxical positive association between built environment and exercise volume among corporate employees and its absence among farmers and migrant workers.

This study offers several notable strengths. First, by situating the analysis within specific occupational contexts, it moves beyond the general-population approach that characterizes much of the prior literature on Chinese women’s physical activity, thereby revealing how exercise participation patterns vary across distinct work environments. Second, it provides empirical evidence corroborating the documented gap in leisure-time exercise between Chinese women and their counterparts in Western developed nations within a concrete occupational setting, strengthening the external validity of previous cross-national observations. Third, the multi-occupational comparative design captures the heterogeneity of exercise behaviors and their perceived barriers among different female worker groups, offering a nuanced evidence base for developing targeted, workplace-specific health promotion interventions. Evertheless, this study is subject to several limitations: it relies on a cross-sectional questionnaire survey, which can only reveal associations rather than causal relationships; furthermore, it suffers from an imbalanced sample structure and measurement bias inherent in self-reported data. Future research should implement longitudinal tracking designs to elucidate the causal pathways between the occupational environment and physical behaviors, and integrate semi-structured mixed-methods approaches to explore the subjective exercise experiences of different groups.

The findings of this study carry meaningful implications for policy and practice. From a policy perspective, the pronounced occupational stratification in physical exercise participation underscores the need for differentiated and compensatory public exercise services. Public-sector employees (civil servants and educators) benefit from institutional resources and organizational mobilization through the “danwei” system, whereas farmers and migrant workers face structural deficits in exercise infrastructure and leisure time. Policies should therefore prioritize resource allocation to rural and migrant populations—including community exercise facilities in urban villages and rural townships—while leveraging workplace platforms to expand organized exercise programs for public-sector employees. From a practical standpoint, the occupation-specific barrier profiles revealed by the stratified regression models indicate that one-size-fits-all interventions are unlikely to be effective. For corporate employees, interventions should target interpersonal barriers and personal perseverance; for migrant workers, time constraints are the primary obstacle, necessitating flexible, low-threshold exercise options; and for civil servants, policy-level barriers warrant institutional reforms that protect exercise time within working hours. The finding that farmers may conflate agricultural labor with structured exercise highlights the importance of public health education that distinguishes occupational physical activity from leisure-time exercise, particularly in rural populations. Future surveys should incorporate objective measurement tools (e.g., accelerometry) to accurately disentangle OPA from LTPA among farming populations, and intervention studies should be conducted to evaluate the effectiveness of occupation-tailored exercise promotion strategies.

## 5 Conclusion

In conclusion, the physical participation patterns of various occupational women in China exhibit cross-group universal commonalities while displaying clear stratification disparities driven by occupational resources and labor modalities. Future public service policies should provide differentiated and compensatory public sports services tailored to the distinct characteristics identified in this study, thereby promoting the equalization of public sports and health services.

## Data Availability

The raw data supporting the conclusions of this article will be made available by the authors, without undue reservation.
