# Supplementary material for: Analysis of the characteristics and differences in exercise participation among seven occupational categories of female workers in China—a cross-sectional survey of 31 provinces across the country
Source: Front Public Health. 2026 Sep 9;14:1923105. doi: 10.3389/fpubh.2026.1923105 (PMC13597825; doi:10.3389/fpubh.2026.1923105)
Supplement: Supplementary file 1 [file Data_Sheet_1.docx]

**Supplementary Table S1. Post-hoc pairwise comparisons (Tukey HSD) of exercise intensity, duration, and frequency across seven occupational groups**

| **Dependent variable** | **Group (I)** | **Group (J)** | **Mean diff. (I−J)** | **SE** | **p** | **95% CI** |
| --- | --- | --- | --- | --- | --- | --- |
| ****Exercise intensity per session**** | Corporate employees | Private business owners | −0.037 | 0.060 | 0.996 | [−0.21, 0.14] |
|  | Corporate employees | Educators | −0.151 | 0.059 | 0.136 | [−0.32, 0.02] |
|  | Corporate employees | Civil servants | −0.027 | 0.039 | 0.993 | [−0.14, 0.09] |
|  | Corporate employees | Farmers | −0.051 | 0.076 | 0.994 | [−0.28, 0.17] |
|  | Corporate employees | Migrant workers | 0.172 | 0.079 | 0.302 | [−0.06, 0.40] |
|  | Corporate employees | Healthcare workers | 0.179 | 0.067 | 0.108 | [−0.02, 0.38] |
|  | Private business owners | Educators | −0.114 | 0.077 | 0.756 | [−0.34, 0.11] |
|  | Private business owners | Civil servants | 0.010 | 0.063 | 1.000 | [−0.18, 0.20] |
|  | Private business owners | Farmers | −0.014 | 0.091 | 1.000 | [−0.28, 0.25] |
|  | Private business owners | Migrant workers | 0.209 | 0.093 | 0.270 | [−0.07, 0.48] |
|  | Private business owners | Healthcare workers | 0.216 | 0.083 | 0.131 | [−0.03, 0.46] |
|  | Educators | Civil servants | 0.124 | 0.062 | 0.406 | [−0.06, 0.31] |
|  | Educators | Farmers | 0.100 | 0.090 | 0.924 | [−0.17, 0.37] |
|  | Educators | Migrant workers | **0.323*** | 0.092 | 0.008 | [0.05, 0.59] |
|  | Educators | Healthcare workers | **0.330*** | 0.082 | 0.001 | [0.09, 0.57] |
|  | Civil servants | Farmers | −0.024 | 0.078 | 1.000 | [−0.25, 0.21] |
|  | Civil servants | Migrant workers | 0.199 | 0.081 | 0.172 | [−0.04, 0.44] |
|  | Civil servants | Healthcare workers | **0.206*** | 0.070 | 0.049 | [0.00, 0.41] |
|  | Farmers | Migrant workers | 0.223 | 0.104 | 0.327 | [−0.08, 0.53] |
|  | Farmers | Healthcare workers | 0.230 | 0.096 | 0.198 | [−0.05, 0.51] |
|  | Migrant workers | Healthcare workers | 0.007 | 0.098 | 1.000 | [−0.28, 0.29] |
| ****Exercise duration per session**** | Corporate employees | Private business owners | −0.060 | 0.099 | 0.997 | [−0.35, 0.23] |
|  | Corporate employees | Educators | 0.002 | 0.096 | 1.000 | [−0.28, 0.29] |
|  | Corporate employees | Civil servants | **−0.193*** | 0.064 | 0.039 | [−0.38, −0.01] |
|  | Corporate employees | Farmers | −0.271 | 0.125 | 0.313 | [−0.64, 0.10] |
|  | Corporate employees | Migrant workers | **−0.587*** | 0.129 | <0.001 | [−0.97, −0.21] |
|  | Corporate employees | Healthcare workers | −0.253 | 0.110 | 0.247 | [−0.58, 0.07] |
|  | Private business owners | Educators | 0.062 | 0.126 | 0.999 | [−0.31, 0.43] |
|  | Private business owners | Civil servants | −0.133 | 0.103 | 0.856 | [−0.44, 0.17] |
|  | Private business owners | Farmers | −0.211 | 0.149 | 0.793 | [−0.65, 0.23] |
|  | Private business owners | Migrant workers | **−0.528*** | 0.152 | 0.010 | [−0.98, −0.08] |
|  | Private business owners | Healthcare workers | −0.193 | 0.137 | 0.797 | [−0.60, 0.21] |
|  | Educators | Civil servants | −0.195 | 0.101 | 0.460 | [−0.49, 0.10] |
|  | Educators | Farmers | −0.273 | 0.147 | 0.514 | [−0.71, 0.16] |
|  | Educators | Migrant workers | **−0.589*** | 0.151 | 0.002 | [−1.03, −0.14] |
|  | Educators | Healthcare workers | −0.255 | 0.135 | 0.491 | [−0.65, 0.14] |
|  | Civil servants | Farmers | −0.078 | 0.128 | 0.997 | [−0.46, 0.30] |
|  | Civil servants | Migrant workers | **−0.394*** | 0.132 | 0.046 | [−0.78, 0.00] |
|  | Civil servants | Healthcare workers | −0.060 | 0.114 | 0.999 | [−0.40, 0.28] |
|  | Farmers | Migrant workers | −0.317 | 0.170 | 0.509 | [−0.82, 0.19] |
|  | Farmers | Healthcare workers | 0.018 | 0.157 | 1.000 | [−0.44, 0.48] |
|  | Migrant workers | Healthcare workers | 0.335 | 0.160 | 0.359 | [−0.14, 0.81] |
| ****Exercise frequency per week**** | Corporate employees | Private business owners | −0.005 | 0.112 | 1.000 | [−0.33, 0.32] |
|  | Corporate employees | Educators | 0.084 | 0.109 | 0.988 | [−0.24, 0.40] |
|  | Corporate employees | Civil servants | **−0.318*** | 0.072 | <0.001 | [−0.53, −0.11] |
|  | Corporate employees | Farmers | **−0.444*** | 0.141 | 0.028 | [−0.86, −0.03] |
|  | Corporate employees | Migrant workers | **−0.751*** | 0.146 | <0.001 | [−1.18, −0.32] |
|  | Corporate employees | Healthcare workers | −0.103 | 0.124 | 0.982 | [−0.47, 0.26] |
|  | Private business owners | Educators | 0.089 | 0.142 | 0.996 | [−0.33, 0.51] |
|  | Private business owners | Civil servants | −0.313 | 0.117 | 0.103 | [−0.66, 0.03] |
|  | Private business owners | Farmers | −0.439 | 0.168 | 0.124 | [−0.93, 0.06] |
|  | Private business owners | Migrant workers | **−0.746*** | 0.172 | <0.001 | [−1.25, −0.24] |
|  | Private business owners | Healthcare workers | −0.098 | 0.155 | 0.996 | [−0.55, 0.36] |
|  | Educators | Civil servants | **−0.402*** | 0.114 | 0.008 | [−0.74, −0.07] |
|  | Educators | Farmers | **−0.527*** | 0.166 | 0.026 | [−1.02, −0.04] |
|  | Educators | Migrant workers | **−0.835*** | 0.170 | <0.001 | [−1.34, −0.33] |
|  | Educators | Healthcare workers | −0.187 | 0.153 | 0.885 | [−0.64, 0.26] |
|  | Civil servants | Farmers | −0.126 | 0.145 | 0.977 | [−0.55, 0.30] |
|  | Civil servants | Migrant workers | −0.433 | 0.150 | 0.058 | [−0.87, 0.01] |
|  | Civil servants | Healthcare workers | 0.215 | 0.129 | 0.640 | [−0.17, 0.60] |
|  | Farmers | Migrant workers | −0.307 | 0.193 | 0.685 | [−0.88, 0.26] |
|  | Farmers | Healthcare workers | 0.340 | 0.177 | 0.465 | [−0.18, 0.86] |
|  | Migrant workers | Healthcare workers | **0.648*** | 0.181 | 0.006 | [0.11, 1.18] |
